# Pedicled buccal fat pad for the augmentation of facial depression deformity: A case report

**DOI:** 10.1097/MD.0000000000007599

**Published:** 2017-07-28

**Authors:** Seiji Komatsu, Kou Ikemura, Yoshihiro Kimata

**Affiliations:** a Department of Plastic and Reconstructive Surgery, Okayama Rosai Hospital; b Department of Plastic and Reconstructive Surgery, Iwate Medical University, Iwate; c Department of Plastic and Reconstructive Surgery, Okayama University Graduate School of Medicine, Dentistry and Pharmaceutical Sciences, Okayama, Japan.

**Keywords:** buccal fat pad, depressed scar, depression deformity, tissue augmentation

## Abstract

**Rationale:**

Tissue augmentation of facial depression deformities can be achieved by volume replacement with autologous fat injection, dermal filler injection, etc. Here, we report a case of tissue augmentation of a facial depression deformity using a pedicled buccal fat pad (BFP).

**Patient concerns:**

A 64-year-old woman was referred with a chief complaint of facial depression deformity.

**Diagnoses:**

Her molars had been removed at another hospital 12 years prior to this referral, and the patient suffered from a left cheek depression deformity as a sequela of a postextraction infection.

**Interventions:**

An incision was made in the left gingivobuccal sulcus under local anesthesia, and BFP was carefully excised from its normal location. The subcutaneous scar tissue was dissected, and a pocket was created via the same mucosal incision. BFP was then pushed into the pocket.

**Outcomes:**

The depression deformity immediately disappeared postoperatively. The transplanted BFP remained unabsorbed and soft 43 months postoperatively. The patient did not have any complications.

**Lessons:**

This novel procedure has 2 advantages. First, the pedicled BFP is a vascularized tissue and is not absorbed postoperatively; control of contour is easy, and only 1 treatment session is required. Complications associated with fat necrosis can be avoided. Second, only a single intraoral incision is required; the risk of donor-site morbidity is very low, and scar formation does not occur on exposed skin. Third, this procedure can be performed without special instruments and equipment. The main disadvantages are limited rotation arc and volume of pedicled BFP. Despite its limited application, this procedure is simple and useful, with low invasiveness.

## 1 Introduction

Tissue augmentation of facial depression deformities can be achieved by volume replacement with an autologous fat injection, dermal filler, and so on.^[1–4]^ Autologous fat injection is widely accepted as biocompatible, nonallergenic, nontoxic, easy to obtain, and being synergistic with the natural skin.^[1]^ However, the rate of long-term survival of fat grafts is unpredictable. Thus, control of contour is difficult and several treatment sessions are often required.^[1,3]^ In addition, complications, such as calcification and oil cyst, secondary to fat necrosis have been frequently reported.^[4]^

Similarly, dermal filler injection is a common procedure for facial augmentation. However, permanent fillers often need removal of the material because of unfavorable results, and temporally fillers need repetitive treatments. Moreover, various complications have been reported after filler injection.^[5]^

We have invented and utilized a buccal fat pad (BFP) for tissue augmentation of a facial depression deformity caused by scar contracture. Here, we report a case of tissue augmentation of a facial depression deformity with a pedicled BFP.

## 2 Case presentation

**Figure 1:**
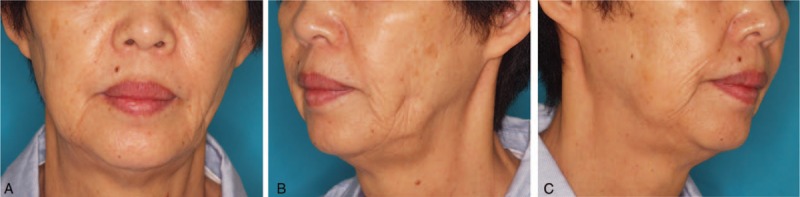
Preoperative view. Depression deformity of the left cheek.

A 64-year-old woman was referred to the Department of Plastic and Reconstructive Surgery, Okayama University Hospital, with a chief complaint of facial depression deformity. Her molars had been removed at another hospital 12 years prior to this referral, and the patient suffered from a left cheek depression deformity as a sequela of a postextraction infection. The depression deformity was 20 mm in diameter and 5 mm in depth (Fig. 1). Her past medical history is significant for rheumatoid arthritis and hypertension.

## 3 Surgical procedure

**Figure 2:**
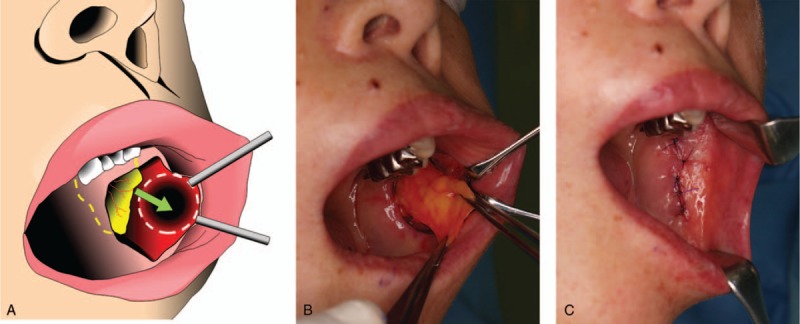
Intraoperative view. The inferior part of the body of BFP was gently dissected from the buccinator medially and the masseter laterally without cutting the connections to 4 extensions. BFP was carefully excised from its normal location using forceps. The pocket was created through the same mucosal incision. BFP was pushed into the pocket without tension. BFP and the buccal mucosa were sutured. BFP = buccal fat pad.

**Figure 3:**
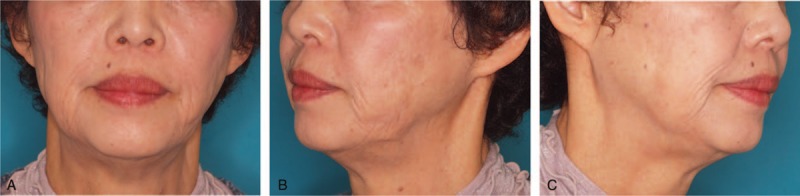
Postoperative view after 6 months.

BFP is a mass of specialized fatty tissue, which is distinct from subcutaneous fat, and consists of a main body and 4 extensions: buccal, pterygoid, superficial, and deep temporal. The body is centrally positioned and is located above the parotid duct and extends along the anterior border of the masseter.^[6]^ A vertical mucosal incision was made in the left gingivobuccal sulcus under local anesthesia. The inferior part of the body was gently dissected from the buccinator medially and the masseter laterally without cutting the connections to 4 extensions. BFP was carefully excised from its normal location using forceps without harming the coating layer of BFP. The subcutaneous scar tissue was then dissected, and a pocket was created through the same mucosal incision. BFP was pushed into this pocket without tension (Fig. 2-1 and 2-2). BFP was not sutured to the dermis to prevent the formation of a new skin dimple, but BFP and the buccal mucosa were sutured using Vicryl (Ethicon, Inc., Somerville, NJ; Fig. 2-3).

This procedure was conducted in compliance with the Declaration of Helsinki. Because this report did not involve any human trials, an ethical review and ethical approval were not necessary. Written informed consent was obtained from the patient for publication of this case report and any accompanying images.

## 4 Results

**Figure 4:**
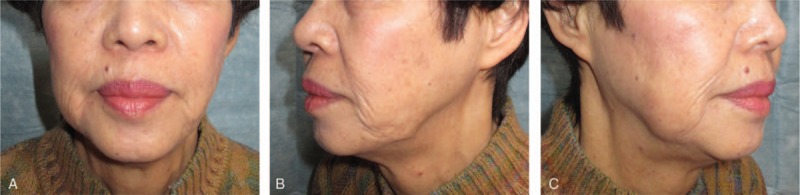
Postoperative view after 43 months. The transplanted BFP remained unabsorbed and soft. BFP = buccal fat pad.

The depression deformity immediately disappeared postoperatively. The transplanted BFP remained unabsorbed and soft at a follow-up 43 months postoperatively (Fig. 3, 4). The patient did not have any complications, such as facial paralysis.

## 5 Discussion

Reconstruction using a pedicled BFP was first reported in 1977.^[7]^ Various reconstructions using BFP have already been reported, but most of these papers describe reconstructions of intraoral defects, such as tumor excision, fistula, and fibrosis.^[6–16]^ Only few reports describe the use of BFP for facial augmentation.^[17–20]^ Reconstruction using BFP after parotidectomy has been reported.^[17]^ In a study, BFP was used for covering the exposed facial nerve to prevent Frey syndrome and to improve the contour of the cheek and retromandibular areas. In addition, orthognathic surgeries using BFP have been reported.^[18–20]^ In these studies, BFP was used to improve the postoperative contour of the lip and malar areas. To the best of our knowledge, this is the first report to describe the reconstruction of a depression deformity due to a scar.

This novel procedure has 3 advantages. First, the pedicled BFP is a vascularized tissue; therefore, it is not postoperatively absorbed. The long-term survival rate of autologous fat injection is improving by technical innovation, although the survival rate is affected by technical factors and unstability.^[1,3]^ Furthermore, complications associated with fat necrosis can sometimes occur.^[1,4]^ BFP has a rich plexus of blood vessels from branches of the maxillary, superficial temporal, and facial arteries that allows it to be used as a pedicled flap.^[19]^ As the pedicled BFP is a vascularized flap that is not absorbed, control of contour is easy and only 1 treatment session is required. Complications associated with fat necrosis can be avoided. There are reports of percutaneous subcision performed for facial depression deformity similar to this case.^[21,22]^ Although percutaneous subcision has low invasiveness and is simple, the results were somewhat unstable, and fat or dermal filler injection were performed in some cases. Especially, when there is a shortage of adipose tissue rather than mere adhesion as in this case, tissue augmentation is necessary. Second, only a single intraoral incision is required. Donor-site morbidity is very low, and scar formation does not occur over the exposed skin. Third, this procedure can be performed without special instruments and equipment. In 2001, Zuk et al^[23]^ reported a study on adipose-derived stem/stromal cells. Clinical applications of adipose-derived stem/stromal cells have been reported. In addition, the effectiveness of platelet-rich plasma and basic fibroblast factor has been reported in the recent years.^[2,24]^ These novel treatments may become the first choice for treating depression deformities in the future but can be performed in only a limited number of institutions because of financial, institutional, and technical problems. In contrast, augmentation with a pedicled BFP can be conducted in any institution.

The main disadvantages of this procedure are limited rotation arc and volume of pedicled BFP. The average volume of BFP is approximately 10 mL, with a mean thickness of approximately 6 mm.^[6]^ When the deformity is severe or distant from BFP, different procedures must be utilized.

Potential complications are minimal. However, hematoma, infection, facial nerve injury, partial necrosis, and excessive scar formation have been reported.^[13]^ Caution in this procedure is necessary to avoid damage to the facial nerve when creating the pocket. However, as the intraoperative visual field is relatively good, the risk of damage to the facial nerve is low.

## 6 Conclusion

We report a new application of BFP, that is, tissue augmentation of a facial depression deformity caused by scar. Although this procedure has limited application, it has low invasiveness and is a simple and useful procedure.
